# A Novel Magnetoelastic Nanobiosensor for Highly Sensitive Detection of Atrazine

**DOI:** 10.1186/s11671-018-2840-7

**Published:** 2018-12-24

**Authors:** Shengbo Sang, Xing Guo, Rong Liu, Jingzhe Wang, Jinyu Guo, Yixia Zhang, Zhongyun Yuan, Wendong Zhang

**Affiliations:** 1 MicroNano System Research Center, Key Lab of Advanced Transducers and Intelligent Control System of the Ministry of Education & College of Information Engineering, Taiyuan University of Technology, Jinzhong, 030600 China; 2 Department of Biomedical Engineering, Shanxi Key Laboratory of Material Strength & Structural Impact, College of Mechanics, Taiyuan University of Technology, Jinzhong, 030600 China

**Keywords:** ME nanobiosensor, ME materials, AuNPs, Atrazine detection

## Abstract

Here, we firstly report a wireless magnetoelastic (ME) nanobiosensor, based on ME materials and gold nanoparticles (AuNPs), for highly sensitive detection of atrazine employing the competitive immunoassay. In response to a time-varying magnetic field, the ME material longitudinally vibrates at its resonance frequency which can be affected by its mass loading. The layer of AuNPs coating on the ME material contributes to its biocompatibility, stability, and sensitivity. The atrazine antibody was oriented immobilized on the AuNPs-coated ME material surface through protein A, improving the nanobiosensor’s performance. Atomic force microscope (AFM) analysis proved that the immobilization of atrazine antibody was successful. Furthermore, to enhance the sensitivity, atrazine–albumin conjugate (Atr–BSA) was induced to compete with atrazine for binding with atrazine antibody, amplifying the signal response. The resonance frequency shift is inversely and linearly proportional to the logarithm of atrazine concentrations ranging from 1 ng/mL to 100 μg/mL, with the sensitivity of 3.43 Hz/μg mL^−1^ and the detection limit of 1 ng/mL, which is significantly lower than the standard established by US Environmental Protection Agency (EPA). The experimental results indicated that the ME nanobiosensor displayed strong specificity and stability toward atrazine. This study provides a new convenient method for rapid, selective, and highly sensitive detection of atrazine, which has implications for its applications in water quality monitoring and other environmental detection fields.

## Introduction

With the rapid development of industry and agriculture, more and more environmental contaminants were released into the ecological environment [1], which caused widespread concern about the relevant researches [2, 3]. In recent years, herbicides have been used in increasing amounts to improve quality and yield in agriculture fields, but many herbicides can remain active in water and soils for years causing serious environmental pollution [4]. Herbicide pollution has attracted considerable attention due to its ecological contamination in water or in agriculture products [5]. Among herbicides, atrazine (2-chloro-4-ethylamino-6-isopropylamino-1, 3, 5-triazine) is the most extensively used for broad-leaf plants and grassy weeds control around the world [6].

Although atrazine has certain inhibitory effect on some perennial weeds, as the environmental contaminant, it is highly toxic [7] and may cause health risks for humans and other animal species [8]. Long-term high concentrations of atrazine intake can impair animal or human health, such as cancer, birth defects, and damage to the heart and liver [9, 10]. The USA, the European Union, and Japan have all included atrazine in the list of endocrine-disrupting chemicals [11]. In the USA, the Environmental Protection Agency (EPA) allows the permissible limit 3 μg/L (Lifetime Health Advisory Level) of atrazine in drinking water [12]. Thus, it is necessary to accurately quantify atrazine at low concentrations.

Many conventional analytical techniques have been developed for atrazine detection, including LC coupled to mass spectrometry (LC–MS) [13], high-performance liquid chromatography (HPLC) [14], and gas chromatography coupled also with mass spectrometry(GC–MS) [15], but these methods also have some limitations, such as high-cost, need of large instruments, poor selectivity, and time-consuming [16].

As a wireless mass-sensitive platform, the magnetoelastic (ME) sensor made from ME material has been widely developed for various applications due to their critical advantages of low cost, high sensitivity, smaller size, and ease of use [17, 18]. Currently, ME sensors are usually made of amorphous ferromagnetic alloys materials, such as Metglas 2826 MB (Fe_40_Ni_38_Mo_4_B_18_) [19]. Under the externally applied alternating and static magnetic fields, the ME material longitudinally vibrates at its resonance frequency [20], generating a magnetic flux density that can be wirelessly detected by a pickup coil without any direct physical connections [21]. According to Eq. (1) [22], the fundamental resonance frequency *f*_0_ depends on the material length *L*, density *ρ*, elastic modulus *E*, and Poisson’s ratio *v*.

1 $$ {f}_0=\frac{1}{2L}\sqrt{\frac{E}{\rho \left(1-{v}^2\right)}} $$

A small additional mass load *∆m* deposited on the ME material surface of mass *M* (*∆m* ≪ *M*) causes a shift in the resonance frequency (*∆f*), which is given by Eq. (2) [23].

2 $$ \frac{\Delta  f}{\Delta  m}=-\frac{f_0}{2M} $$

Based on above unique properties of the ME material, the resonance frequency of the ME material decreases with an increase of the extra mass load. Thus, through their functionalization with a sensing film, the ME materials have been developed for physical, chemical, and biological analysis, such as the detection of stress/pressure [24], temperature/humidity [25], carbon dioxide [26], endotoxin [27], Salmonella typhimurium/*Bacillus anthracis* spores [28], and *Escherichia coli* O157:H7 [29]. To our knowledge, however, no application of the ME material has been applied on the atrazine detection.

In this research, utilizing its excellent properties and advantages, we firstly proposed a wireless ME nanobiosensor employing the ME material as the substrate and gold nanoparticles (AuNPs) as the coating layer, for atrazine detection at ppb level on the basis of the direct competitive immunoassay procedures. Compared with the covalent-random antibody immobilization, the covalent-oriented strategy is more beneficial to improve the sensitivity of the nanobiosensor. Because the protein A is an interesting alternative to specifically bind with the Fc immunoglobulin region of the antibody, it was employed for oriented immobilization of the atrazine antibody [30], giving the highest immobilization density, to exhibit better antigen binding efficiency and improve nanobiosensor’s performance [31]. The direct competitive immunoassay for atrazine was constructed by oriented immobilization of atrazine antibody to protein A covalently modified on the AuNPs-coated ME material surface, followed by the competitive reaction of atrazine–albumin conjugate (Atr–BSA) and atrazine with the atrazine antibody. Atr–BSA was induced to amplify the signal responses, in turn significantly increasing the sensitivity of the nanobiosensor. The efficiency of the ME nanobiosensor was evaluated, demonstrating that a novel ME nanobiosensor for the detection of trace concentrations of atrazine was successfully developed.

## Materials and Methods

### Materials

Atrazine antibody, atrazine–albumin conjugate antigen (Atr–BSA), atrazine, and protein A were purchased from EastCoast Bio (Maine, USA). Simazine, prometryn, and dichlorodiphenyltrichloroethane (DDT) were obtained from Chengdu Huaxia Chemical Reagent Co., Ltd. Cysteamine, 1-ethyl-3-(3-dimethylaminopropyl) carbodiimide hydrochloride (EDC), *N*-hydroxysulfosuccinimide (NHS), bovine serum albumin (BSA, 99%), and phosphate-buffered saline(PBS buffer, pH = 7.4) were purchased from Sigma-Aldrich Corporation (Saint Louis, MO, USA).

### ME Nanobiosensor Fabrication

#### Preparation of the ME Nanosensor Platform

**Fig. 1:**
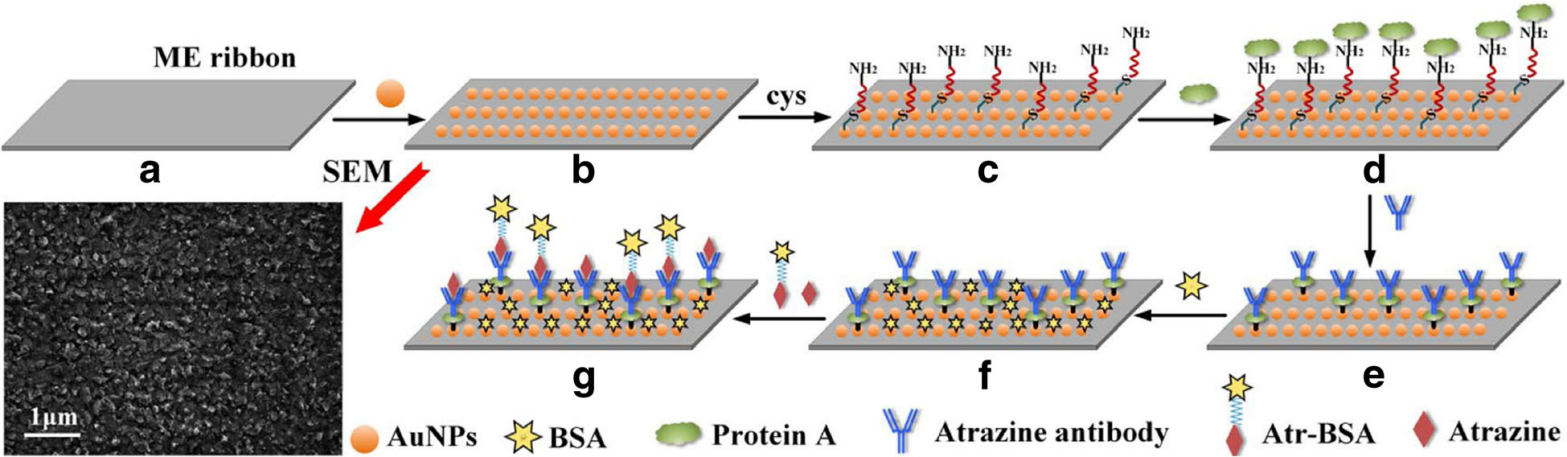
The schematic representation of the procedures of the ME nanobiosensors functionalization: (**a**) the bare ME ribbon; (**b**) the AuNPs coating; (**c**) the SAM layer; (**d**) the protein A immobilization; (**e**) the antibody modification; (**f**) BSA blocking; (**g**) atrazine and Atr–BSA competitively combined with the antibody; SEM image of the AuNPs-coated nanosensor surface

ME material ribbons composed of Metglas alloy 2826 (Fe_40_Ni_38_Mo_4_B_18_) were purchased from Honeywell Corporation (Morristown, NJ,USA) and cut into 5 mm × 1 mm × 0.028 mm using a computer-controlled laser cutting machine. To remove organic film and debris, the ME ribbons were ultrasonically cleaned in acetone and ethanol each for 10 min and rinsed in deionized water, then dried in a stream of nitrogen (Fig. 1a). A ~ 100-nm-thick layer of chromium nanoparticles was sputtered on both sides of the ME ribbon surface to enhance the adhesion between the AuNPs and the ribbon surface. Subsequently, both sides of the chromium-coated ME ribbon surface were sputtered with AuNPs to improve the biocompatibility and protect the ribbon from oxidation and corrosion. The scanning electron microscope (SEM) image in Fig. 1 showed that the AuNPs coated on the ME ribbon were in spherical size. AuNPs and -SH can easily form the Au-S bond. Besides due to its attractive advantages of low-price, non-corrosion, biocompatibility, and nontoxicity [32], AuNPs can provide an excellent interface for the chemical or bio-recognition elements modification [33, 34]. Afterwards, the ME ribbons were annealed in a vacuum oven at 200 °C for 2 h to relieve residual internal stress and promote the adhesion of the AuNPs layer to the ME ribbons. Then, the ME nanosensor platforms were finished and ready for atrazine antibody immobilization (Fig. 1b).

#### Atrazine Antibody Immobilization

The AuNPs-coated nanosensor platforms were ultrasonically cleaned with acetone, isopropanol, deionized water, and ethanol each for 5 min, and dried under a stream of nitrogen. Then, the nanosensor platforms were immersed into cysteamine solution (10 mM) for 12 h at room temperature to obtain a self-assembled monolayer (SAM) (Fig. 1c). The protein A (1 mg/mL) was activated with 4 mg/mL EDC-4 mg/mL NHS for 30 min at room temperature. After that, the activated protein A was incubated on the SAM-modified nanosensors for 30 min at 37 °C and rinsed with PBS buffer (Fig. 1d). The nanosensor platforms were then incubated with atrazine antibody for 50 min and washed with PBS buffer (Fig. 1e). To prevent non-specific adsorption, the atrazine antibody-coated nanosensors were further treated with 0.5% BSA for 30 min, and then rinsed with PBS buffer to remove any unbound BSA and dried under a nitrogen stream. Finally, the ME nanobiosensors were fabricated for atrazine detection (Fig. 1f).

### Signal Measurement

**Fig. 2:**
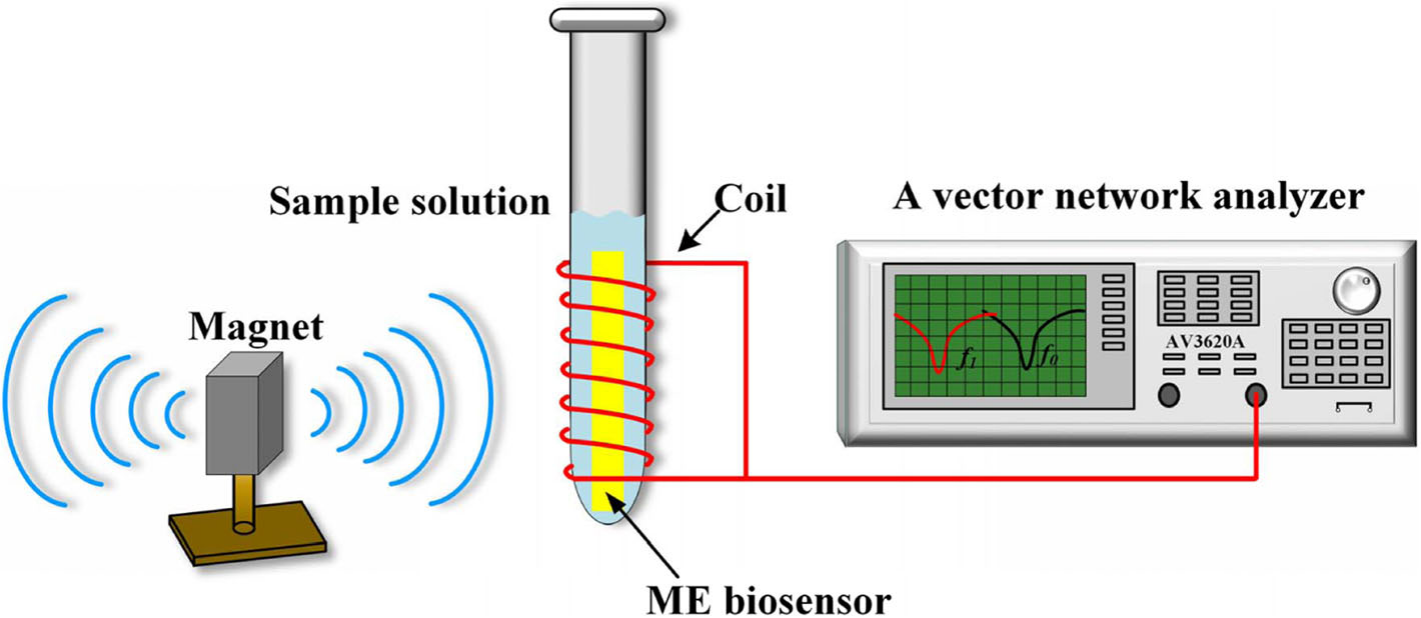
The schematic representation of the wireless ME nanobiosensor measurement system

The resonance frequency of the ME nanobiosensor was measured using a pickup coil wound around a vial, together with a vector network analyzer (AV3620A, the 41st Institute of CETC, Qingdao, China), as schematically represented in Fig. 2. To generate an alternating magnetic field, the network analyzer connected with the pickup coil was operated in the S_11_ mode for providing a swept frequency signal to the coil, and it can monitor the reflected signal from the coil. Additionally, a static magnetic field generated by a bar magnet was applied to enhance the resonance behavior. The nanobiosensors were vertically and wirelessly (without any wire connections with measurement system) inserted into the vial containing 30 μL sample solutions to be tested. All experiments were conducted at room temperature (25 ± 2 °C) in PBS (0.1 M, pH 7.4) solvent system. The resonance frequency of the nanobiosensor can be determined through the measurement of the S_11_ parameter, which was monitored and recorded every 5 min.

## Results and Discussion

### Characterization of the Nanobiosensor Surface Morphology

**Fig. 3:**
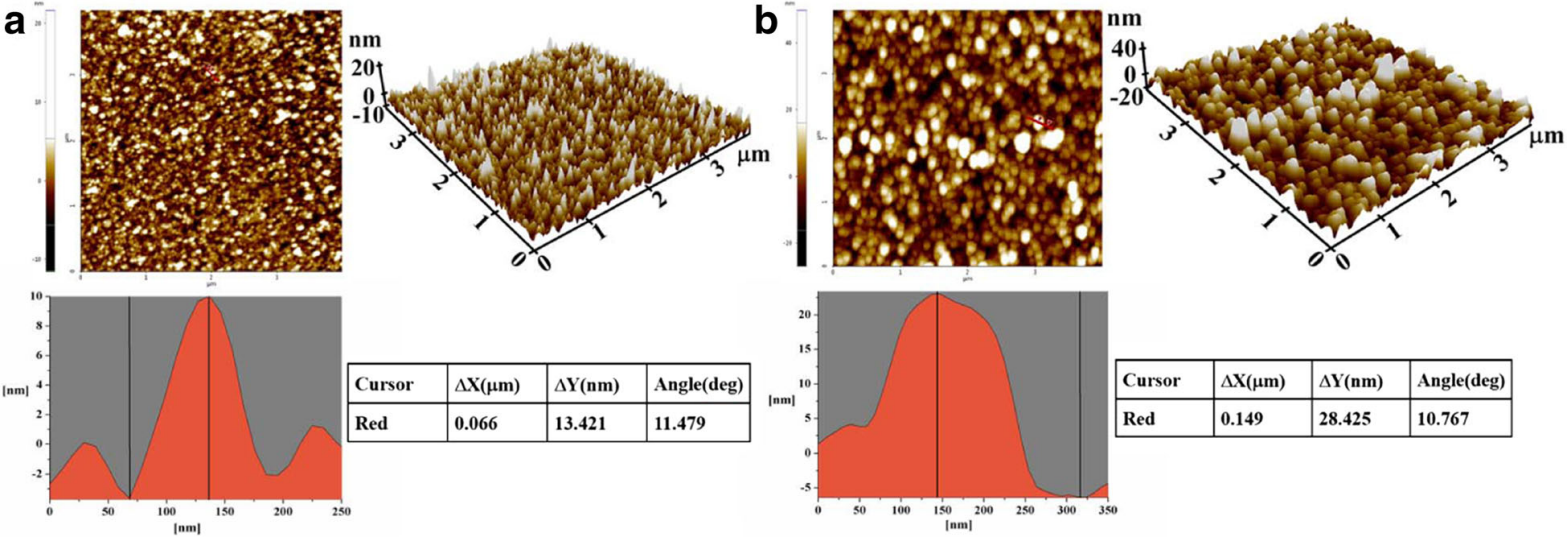
AFM images of (**a**) the AuNPs-coated and (**b**) antibody-modified nanobiosensor surface

Atomic force microscope (AFM, ND-100, Park System, Korea) observation of the nanobiosensor surface was performed to examine the immobilization effect of atrazine antibody. AFM images of the AuNPs-coated nanobiosensor and antibody-modified nanobiosensor surface are presented in Fig. 3a, b, respectively. It is clear that the increased surface roughness results from the covalently immobilized atrazine antibody. Comprehensive analysis of the AFM cross-sections topography shows that the AuNPs-coated nanobiosensor has the height variation of 13.421 nm; however, the value increased to 28.425 nm after the antibody modification. As well known that the diameter of the antibody molecular is about 15 nm, it is obviously concluded that the immobilization of atrazine antibody is successful.

### Optimization for Concentration of Atrazine Antibody

**Fig. 4:**
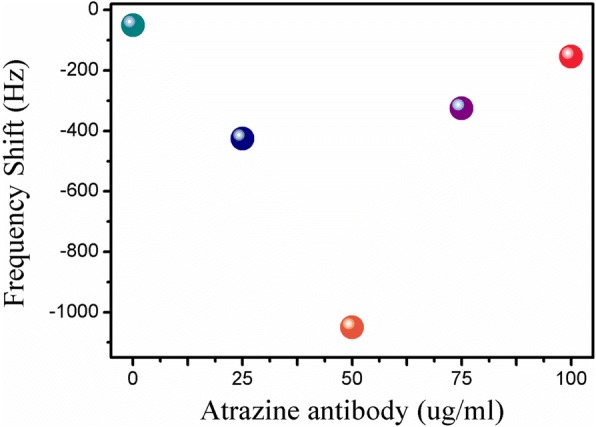
The ME nanobiosensor’s frequency response with different immobilization concentrations of atrazine antibody (0 μg/mL, 25 μg/mL, 50 μg/mL, 75 μg/mL, 100 μg/mL)

The immobilization concentration of the antibody has an important influence on the sensitivity of the nanobiosensor. Therefore, it was necessary to evaluate the resonance frequency response of the nanobiosensor with different immobilization concentrations of atrazine antibody (25 μg/mL, 50 μg/mL, 75 μg/mL, 100 μg/mL). From Fig. 4, we can see that the resonance frequency shift reached maximum at 50 μg/mL. When the concentration of atrazine antibody went up to 75 μg/mL, the response began to decline due to the steric hindrance and the electrostatic repulsion [35]. That is to say, the 50 μg/mL atrazine antibody can attain relatively saturated immobilization. Thus, 50 μg/mL was the optimum concentration of atrazine antibody for immobilization.

### Optimization for Concentration of Atr–BSA

**Fig. 5:**
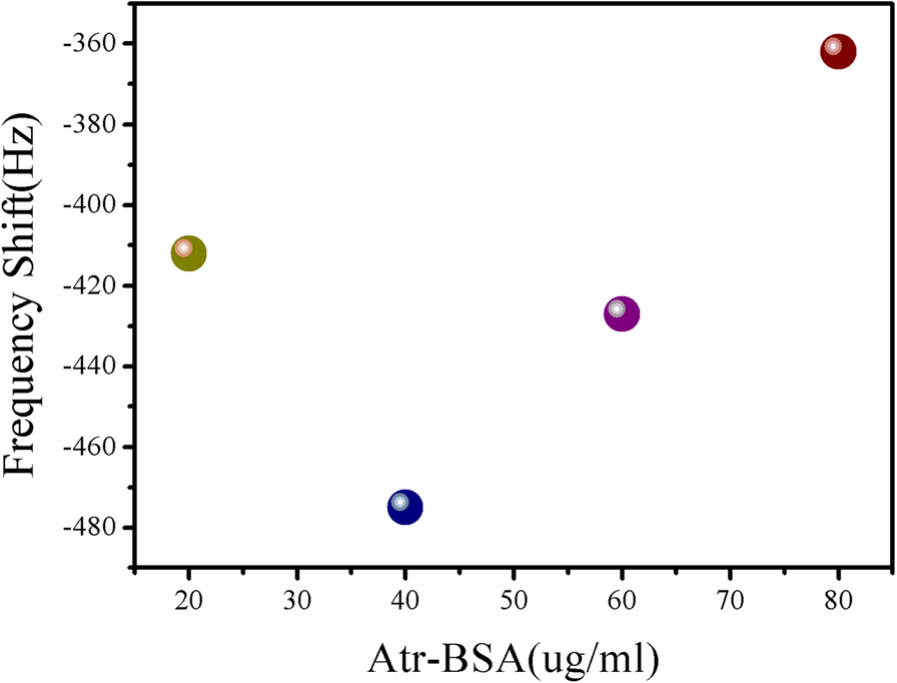
The ME nanobiosensor’s frequency response to Atr–BSA of different concentrations (20 μg/mL, 40 μg/mL, 60 μg/mL, 80 μg/mL)

In the immunoreaction, atrazine and Atr–BSA competed for the limited number of atrazine antibody sites on the nanobiosensor surface. Hence, with the optimum concentration of atrazine antibody for immobilization, the working concentration of Atr–BSA, as an important factor, affects the sensitivity of the nanobiosensor. The optimization process was investigated by determining the ME nanobiosensor’s resonance frequency response to Atr–BSA of different concentrations (20 μg/mL, 40 μg/mL, 60 μg/mL, 80 μg/mL). As indicated in Fig. 5, the maximum response was observed at 40 μg/mL. So 40 μg/mL Atr–BSA was used in the following determination.

### Atrazine Detection

**Fig. 6:**
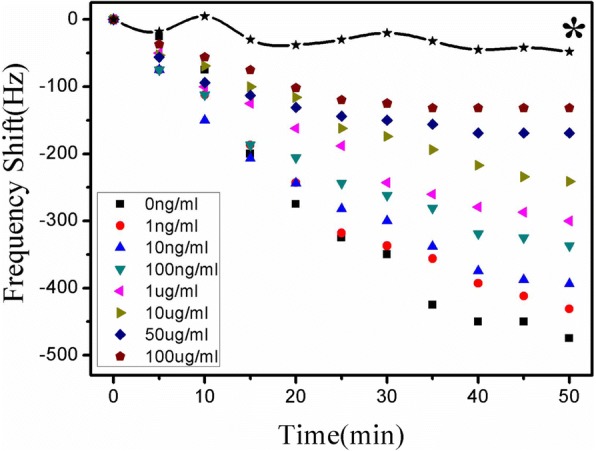
Real-time frequency responses at different atrazine concentrations ranging from 0 to 100 μg/mL. * The blank control response (without atrazine antibody immobilization) to Atr–BSA

Figure 6 depicts the real-time frequency response of the ME nanobiosensor measured in the sample mixture of 15 μL Atr–BSA (40 μg/mL) and 15 μL atrazine with different concentrations (0 ng/mL, 1 ng/mL, 10 ng/mL, 100 ng/mL, 1000 ng/mL, 10 μg/mL, 50 μg/mL, 100 μg/mL). As shown in Fig. 1g, atrazine and Atr–BSA competitively combined with the antibody immobilized on the nanobiosensor surface, which in turn leads to an increase of the mass load on the nanobiosensor surface, consequently causing a decrease of the resonance frequency with the incubation time. It is clear from Fig. 6 that the steady-state response is generally achieved at about 50 min. Atr–BSA concentration and the number of atrazine antibody sites were fixed, so the amount of bonded Atr–BSA on the nanobiosensor were inversely proportional to the concentration of atrazine in solution. The molecular weight of Atr–BSA is greater than that of atrazine. Therefore, the resonance frequency of the nanobiosensor changes inversely with the concentration of atrazine in solution. As shown in Fig. 6, the rate and magnitude of the resonance frequency shift decreased with increasing atrazine concentrations, and a higher concentration of atrazine can induce a smaller resonance frequency shift. Figure 6 curve * represents a background response of the blank control sensor (without atrazine antibody immobilization) to Atr–BSA, which is approximately 48 Hz far less than the detection signal, indicating that the non-specific adsorption can be ignored. Thus, the atrazine concentration can be detected through the resonance frequency shift of the wireless ME nanobiosensor, with an inversely proportional relation.

**Fig. 7:**
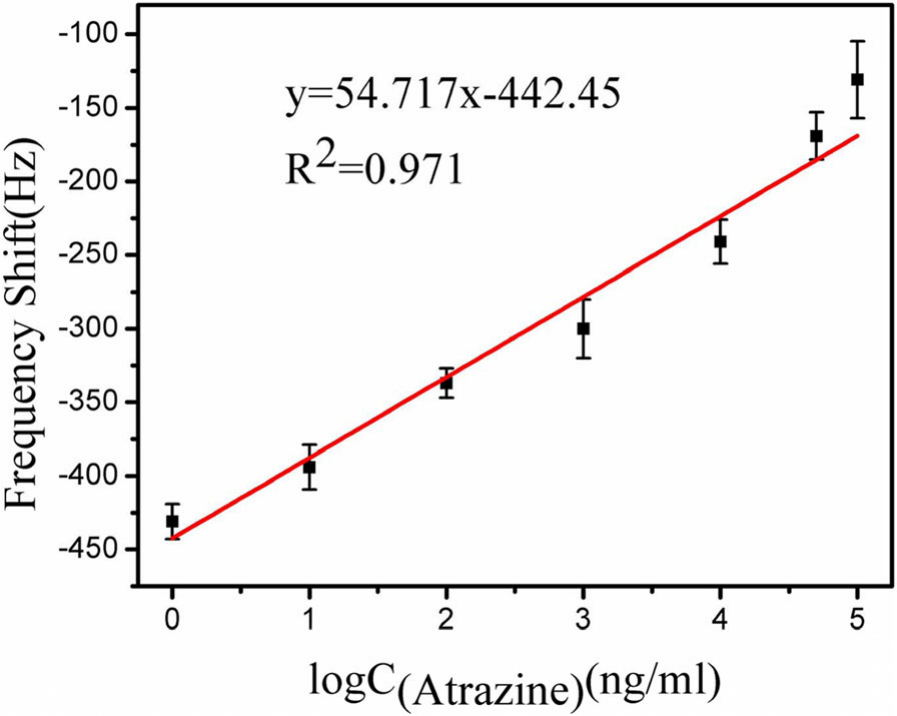
Calibration curve: the 50 min shift in resonance frequency as a function of different atrazine concentrations

The standard calibration curve for the detection of atrazine on the ME nanobiosensor during the first 50 min are shown in Fig. 7. For each concentration, the nanobiosensor calibration experiments were conducted five times under identical conditions. It is found that the resonance frequency shift is linear with the logarithm value of the atrazine concentrations ranging from 1 ng/mL to 100 μg/mL, which can be represented by *∆f* = 54.717 log *C*_Atrazine_ − 442.45 (*R*^2^ = 0.971). The sensitivity is calculated to be 3.43 Hz/μg mL^−1^. It is evident from Fig. 7 that the limit of detection (LOD) is 1 ng/mL, which is significantly lower than the upper permissible limit for atrazine of 3 μg/L given in the US EPA, satisfying the standard currently available. Besides, the detection limit is evidently lower than that of the previous reported methods [36, 37]. It has been demonstrated that a low-cost, wireless, and highly sensitive nanobiosensor was successfully established for real-time detection of atrazine.

Since atrazine is the small molecule, direct competitive immunoassay approach was employed to improve the sensitivity of the ME nanobiosensor. In the direct competitive immunoassay, the antibody is modified on the sensor surface and the signal response results from the binding of Atr–BSA molecule. Conversely, in the indirect competitive immunoassay, Atr–BSA is immobilized on the sensor surface and the response results from the binding of antibody molecule. According to the literature researches [38] and our results, the direct competitive immunoassay is feasible for small molecules monitoring. The indirect competitive immunoassay is highly sensitive to the analyte sample with trace concentration [39]. Although the indirect competitive immunoassay has a higher sensitivity [40, 41], it may be complicated to operate and difficult to implement for repeated reliable use [36]. However, the direct competitive immunoassay is very fast, simple to use, and self-contained—no additional reagents needed [36]. Thus, for the future development, the direct competitive immunoassay may be the most promising method.

### ME Nanobiosensor Specificity

**Fig. 8:**
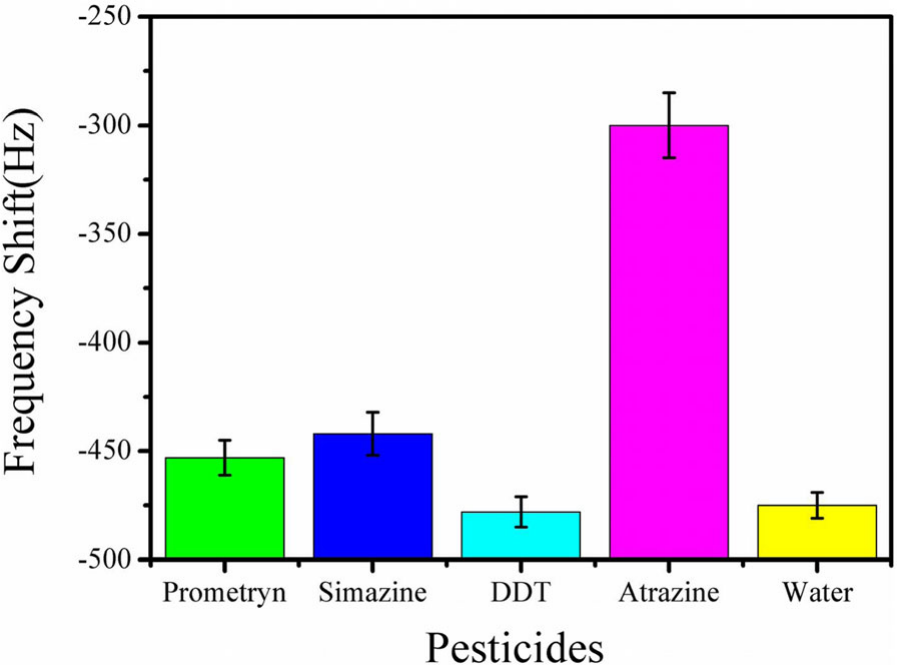
Resonance frequency response of the ME nanobiosensor to other interferents with the concentration of 100 μg/mL

The ME nanobiosensor specificity to atrazine was investigated by determining the nanobiosensor’s responses to some other pesticides, such as prometryn, simazine, and DDT, as shown in Fig. 8. It was evident from Fig. 8 that the ME nanobiosensor showed little responses to these interferences due to non-specific absorption, and the responses to prometryn and simazine were slightly larger than DDT, which had a similar response level to the blank solution. It may be due to the fact that both prometryn and simazine have the similar structures with atrazine, belonging to triazine pesticides; however, DDT is a kind of organochlorine insecticides. The results indicated that atrazine was effectively recognized and specifically combined with the antibody immobilized on the nanobiosensor surface. Thus, the ME nanobiosensor showed strong specificity for atrazine detection.

### ME Nanobiosensor Stability

**Fig. 9:**
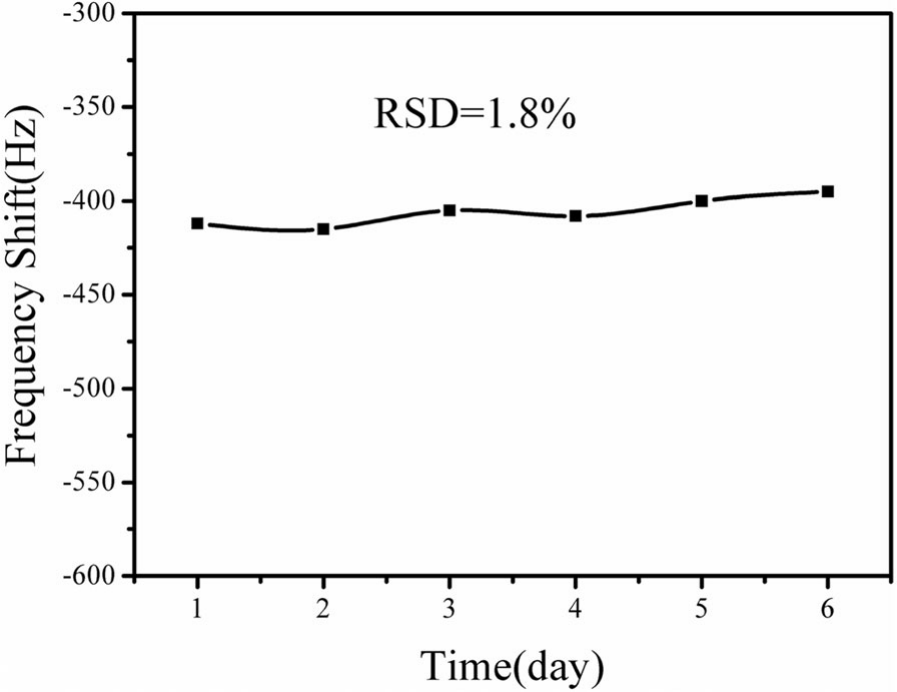
Stability measurements of 10 ng/mL atrazine on the ME nanobiosensor

Figure 9 shows the stability of the ME nanobiosensor toward atrazine detection. Six of the same ME nanobiosensors were prepared and stored in 4 °C, every which was tested towards10 ng/mL atrazine every other day until 6 days. Every single detection cycle only tested one nanobiosensor for 50 min. It is clear that the resonance frequency responses of the nanobiosensors remain nearly constant and the relative standard deviation (RSD) is calculated to be 1.8%. The result demonstrates that the ME nanobiosensor exhibits excellent stability for atrazine detection.

## Conclusions

A wireless ME nanobiosensor based on ME materials and AuNPs was successfully developed for real-time and highly sensitive detection of atrazine employing the competitive immunoassay. The oriented immobilization of atrazine antibody through protein A improved the nanobiosensor’s performance. Atr–BSA with heavy molecule mass and atrazine competitively combined with atrazine antibody on the nanobiosensor surface, amplifying the signal responses, which in turn improved the sensitivity. The resonance frequency shift mainly induced by the bound Atr–BSA is inversely proportional to the target atrazine concentration. Besides, the working concentrations of atrazine antibody and Atr–BSA were optimized to be 50 μg/mL and 40 μg/mL, respectively. Under the optimum conditions, the ME nanobiosensor displays widely linear determination ranges for atrazine from 1 ng/mL to 100 μg/mL, with the satisfactory sensitivity of 3.43 Hz/μg mL^−1^ and the detection limit of 1 ng/mL which is sufficient for the legislative requirements and is lower than other reported methods. AFM images verified that the atrazine antibody was successfully immobilized on the nanobiosensor surface in an oriented manner. The experimental results demonstrate that the ME nanobiosensor has high specificity and stability toward atrazine. Benefiting from its effects on detection limits, simplicity, disposable property, and wireless nature, the study not only proposed a new method for highly sensitive detection of atrazine but also indicated its potential practicability for other environmental contaminants detection and water quality monitoring.

## Abbreviations

AFM: Atom force microscope
Atr–BSA: Atrazine–albumin conjugate antigen
AuNPs: Gold nanoparticles
BSA: Bovine serum albumin
DDT: Dichlorodiphenyltrichloroethane
EDC: 1-Ethyl-3-(3-dimethylaminopropyl) carbodiimide hydrochloride
ME: Magnetoelastic
NHS: *N*-Hydroxysulfosuccinimide
PBS: Phosphate-buffered saline
SAM: Self-assembled monolayer
SEM: Scanning electron microscope
US EPA: Environmental Protection Agency in the United States
